# Incidence of Lyme Borreliosis in the Dutch General Practice Population: A Large-Scale Population-Based Cohort Study Across the Netherlands Between 2015 and 2019

**DOI:** 10.1089/vbz.2022.0048

**Published:** 2023-04-12

**Authors:** Eline Houben, Hilda de Jong, Fernie Penning-van Beest, Josephina Kuiper, Emily Holthuis, Maxim Blum, Jozica Skufca, Margarita Riera-Montes, Bradford D. Gessner, Andreas Pilz, Andrew John Vyse, Elizabeth Begier, Mendwas Dzingina, Ron Herings, James H. Stark

**Affiliations:** ^1^ PHARMO Institute for Drug Outcomes Research, Utrecht, The Netherlands.; ^2^ P95 Pharmacovigilance and Epidemiology, Leuven, Belgium.; ^3^ Pfizer, Inc., Vaccines Medical Development and Scientific and Clinical Affairs, Collegeville, Pennsylvania, USA.; ^4^ Pfizer Corporation, Vaccines Medical Development and Scientific and Clinical Affairs, Wien, Austria.; ^5^ Pfizer UK Ltd., Walton Oaks, United Kingdom.; ^6^ Pfizer, Inc., Patient Health & Impact, Chief Business Office, Pfizer, New York, New York, USA.; ^7^ Department of Epidemiology & Data Science, Amsterdam Public Health Research Institute, Amsterdam UMC, Amsterdam, The Netherlands.

**Keywords:** Lyme borreliosis, incidence rate, Europe, Netherlands, erythema migrans, disseminated Lyme borreliosis

## Abstract

**Background:**

There is a need for updated incidence rates (IRs) of Lyme borreliosis (LB) in Europe, including the Netherlands. We estimated LB IRs stratified by geographic area, year, age, sex, immunocompromised status, and socioeconomic status (SES).

**Methods:**

All subjects registered in the PHARMO General Practitioner (GP) Database without prior diagnosis of LB or disseminated LB and having ≥1 year of continuous database enrolment were included. IRs and corresponding confidence intervals (CIs) of GP-recorded LB, erythema migrans (EM), and disseminated LB were estimated during the period 2015‒2019.

**Results:**

We identified 14,794 events (suspected, probable, or confirmed) with a diagnostic code for LB that included 8219 with a recorded clinical manifestation: 7985 (97%) with EM and 234 (3%) with disseminated LB. National annual LB IRs were relatively consistent, ranging from 111 (95% CI 106‒115) in 2019 to 131 (95% CI 126‒136) in 2018 per 100,000 person-years. Incidence of LB showed a bimodal age distribution, with peak IRs observed among subjects aged 5‒14 and 60‒69 years in men and women. Higher LB incidence was found in subjects who were residents of the provinces of Drenthe and Overijssel, immunocompromised, or of lower SES. Similar patterns were observed for EM and disseminated LB.

**Conclusions:**

Our findings confirm that LB incidence remains substantial throughout the Netherlands with no indication of decline in the past 5 years. Foci in two provinces and among vulnerable populations suggest potential initial target groups for preventive strategies such as vaccination.

## Introduction

Lyme borreliosis (LB) is an infectious disease caused by the bacterium *Borrelia burgdorferi sensu* lato, which is transmitted through infected *Ixodes* tick bites, primarily *Ixodes ricinus* ticks in Europe. Each year ∼1.5 million tick bites occur in the Netherlands, specifically between March and October (National Institute for Public Health and the Environment [RIVM] 2020). Approximately 15% of the ticks are infected with *B. burgdorferi sensu* lato (Hartemink et al. 2021). The risk of contracting LB following a tick bite has been estimated at 2 to 3 per 100 but can be higher when infected ticks feed for prolonged periods (National Institute for Public Health and the Environment [RIVM] 2020). Borrelia-infected ticks are often found in dunes and forests in the Netherlands providing favorable conditions for ticks (Wielinga et al. 2006, Gassner et al. 2011, Hofhuis et al. 2013, 2016). As such, understanding the burden of LB in the Netherlands remains a public health priority.

Although an initiative has been launched to report tick bites and LB in the Netherlands (tekenradar.nl 2012), reporting of LB is not statutorily required. However, general practitioners (GPs) are required to maintain medical records, thereby enabling LB case reporting. Multiple studies have evaluated the burden of LB in the Netherlands over the past several decades (den Boon et al. 2004, Hofhuis et al. 2006, 2015a, 2015b, 2016). Specifically, a survey of GPs in 2017 estimated the incidence of erythema migrans (EM) as 140 and 149 per 100,000 in 2014 and 2017, respectively (van den Wijngaard et al. 2019). Because neither a robust surveillance system based on routine data collection nor recent population-based studies quantifying disease burden are available, understanding the local risk of LB in recent years has not been feasible.

A clear understanding of recent epidemiology is needed for public health officials to effectively implement preventive measures, such as vaccination, targeting endemic areas, and vulnerable populations. This study therefore provides a recent and comprehensive overview of LB epidemiology in the Netherlands based on a large population-based cohort of subjects registered with a GP. Results include analyses of geographic endemicity and incidence within subgroups defined by demographics as well as immunocompromised and socioeconomic status (SES).

## Methods

### Data source and setting

The PHARMO Database Network provides comprehensive information regarding the complete patient journey and health care in the Netherlands. Full details of the PHARMO Database Network are described elsewhere (Kuiper et al. 2020). Briefly, the PHARMO Database Network is a population-based network of electronic health care databases that combine de-identified data from primary and secondary health care settings in the Netherlands. One of these databases is the GP Database, which comprises data from electronic patient records registered by GPs that include information regarding diagnoses and symptoms, laboratory test results, referrals to specialists, and health care product/drug prescriptions. The prescription records include information regarding product type, prescription date, strength, dosage regimen, quantity, and route of administration.

Diagnoses and symptoms are coded according to the International Classification of Primary Care (ICPC), which can be mapped to *International Classification of Diseases, Tenth Revision* codes, but can also be entered as free text. Drug prescriptions are coded according to the World Health Organization Anatomical Therapeutic Chemical Classification System. The nature of the Dutch health care system designates the GP as a gatekeeper; all inhabitants, regardless of age or insurance, are registered at a GP. The GP Database includes data from ∼20% of the Dutch population because not all practices are included (Kuiper et al. 2020). Included practices are spread across the country and data have been shown to be representative of the Dutch population in terms of demographic characteristics, medication use, and diagnoses (Overbeek et al. 2022).

Because all patient-level data in the PHARMO Database Network were de-identified, use for health services research was fully compliant with applicable laws and, accordingly, institutional review board/ethical approval was not needed. The study was conducted in accordance with legal and regulatory requirements, as well as with scientific purpose, value, and rigor and followed generally accepted research practices.

### Study population

All subjects who were registered with a GP between January 1, 2015, and December 31, 2019, were included. The study start date was defined as the first date that subjects had ≥1 year of continuous electronic patient data. Subjects with a diagnosis of LB or EM in the previous 12 months before start of follow-up, as well as all subjects with a diagnosis of disseminated LB any time before start of follow-up, were excluded. Included subjects were followed until end of data collection, death, or December 31, 2019 (study end date), whichever came first.

### Study outcomes

For assessing LB events, all subjects with a diagnostic ICPC code, free-text annotation, or laboratory test result (see Supplementary Table S1 for included diagnostic codes and search terms and Supplementary Table S2 for laboratory tests) for LB, EM, or disseminated LB were considered to have met case definitions. Disseminated LB included Lyme neuroborreliosis (LNB), Lyme arthritis (LA), and other manifestations (acrodermatitis chronica atrophicans [ACA], borrelial lymphocytoma, lymphocytoma cutis [LC], and multiple EM). Case definitions of LB, EM, and disseminated LB were applied and included the categories of suspected, probable, or confirmed (Supplementary Table S1).

The index date was defined as the date when a patient first met any of the case definitions for LB, EM, or disseminated LB. For disseminated LB in particular, given the chronic nature of some conditions, only the first record of each patient contributed to incidence rate (IR) calculations. However, LB and EM recurrences during follow-up were included if they occurred following a washout period of 365 days, during which reported events were considered to belong to the previous event.

### Characteristics

Subjects' characteristics as measured at index date included geographic area, sex, and age (in 10-year age categories for subjects ≥20 years of age and 5-year age categories for subjects <20 years of age). Geographic area was defined by the following provinces of the Netherlands: Drenthe, Flevoland, Friesland, Gelderland, Groningen, Limburg, Noord-Brabant, Noord-Holland, Overijssel, Utrecht, Zeeland, and Zuid-Holland.

Additionally, SES and subjects' immunocompromised status were assessed. SES was categorized as low, middle, high, and unknown and was based on 4-digit zip codes and neighborhood scores from the Netherlands Institute for Social Research that account for income, education, and employment (Knol 2012). Subjects were considered immunocompromised if they had any diagnostic code or free-text annotation of or a prescription for the following conditions within 1 year before start of follow-up: end-stage renal disease, malignant neoplasm, history of bone marrow transplant, spleen anomalies, acquired immunodeficiency syndrome (AIDS), rheumatoid disorders, and immunodeficiency syndrome (see Supplementary Table S3 for algorithms used to identify these conditions) (Chinen and Shearer 2010).

### Statistical analysis

The person-time at risk was estimated from the start of follow-up until the earliest index date or end of follow-up. In situations of LB or EM recurrences, the 365-day washout period was subtracted from the person-time at risk. Where relevant, the person-time at risk was stratified according to age group and year. The IRs of LB (number of LB events per 100,000 person-years) were estimated overall and stratified by sex, age, and geographic area (*i.e.*, provinces) in the Netherlands. Furthermore, stratifications by SES and immunocompromised status were performed and compared by Poisson regression tested at *p* < 0.05; corresponding 95% confidence intervals (95% CIs) were calculated using the Exact Poisson method. All data were analyzed using SAS programs organized within SAS Enterprise Guide version 8.2 (SAS Institute, Inc., Cary, NC) and conducted under Windows using SAS version 9.4.

## Results

Overall, we identified 14,794 GP-recorded events of LB (suspected, probable, or confirmed) in the PHARMO GP Database (*N*_at risk_ = 2,826,858 subjects) during the study period. Of these, 52% were women and the mean age at diagnosis was 47 years (standard deviation, 21 years). Of the 14,794 LB events identified, 8219 had a GP-recorded manifestation of LB, of which 7985 (97%) were classified as EM and 234 (3%) as disseminated LB. The latter comprised 94 events (40%) with a GP record of LNB, 69 (29%) with LA, and 71 (30%) with another manifestation (47 [66%] ACA, 17 [24%] borrelial lymphocytoma, 4 [6%] multiple EM, and 3 [4%] LC). Events of LB, EM, and disseminated LB, including LNB, LA, and other manifestations, are displayed by level of diagnostic certainty in Supplementary Fig. S1.

**FIG. 1.**
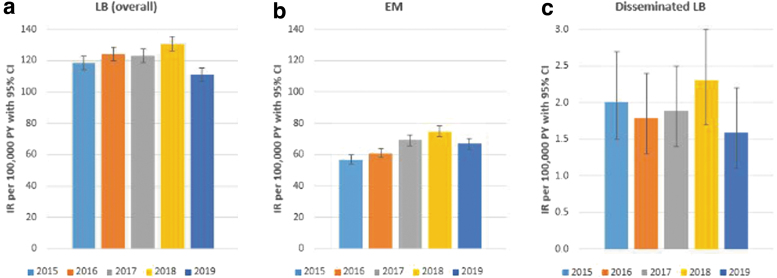
Incidence rate of GP-recorded LB and its manifestations in the Netherlands, stratified by year: **(a)** LB overall; **(b)** EM; **(c)** disseminated LB. EM, erythema migrans; GP, general practitioner; IR, incidence rate; LB, Lyme borreliosis; PY, person-year.

The estimated IRs of LB, based on either suspected, probable, or confirmed events of LB, were similar throughout the individual study years and ranged from 111 (95% CI 106–115) in 2019 to 131 (95% CI 126–136) per 100,000 person-years in 2018. For EM, annualized IRs varied from 57 (95% CI 54–60) in 2015 to 75 (95% CI 71–78) per 100,000 person-years in 2018, whereas annualized IRs for disseminated LB ranged from 1.6 (95% CI 1.1–2.2) per 100,000 person-years in 2019 to 2.3 (95% CI 1.7–3.0) in 2018 (Fig. 1). Most LB events occurred in June (18%), July (20%), and August (17%; Supplementary Fig. S2).

**FIG. 2.**
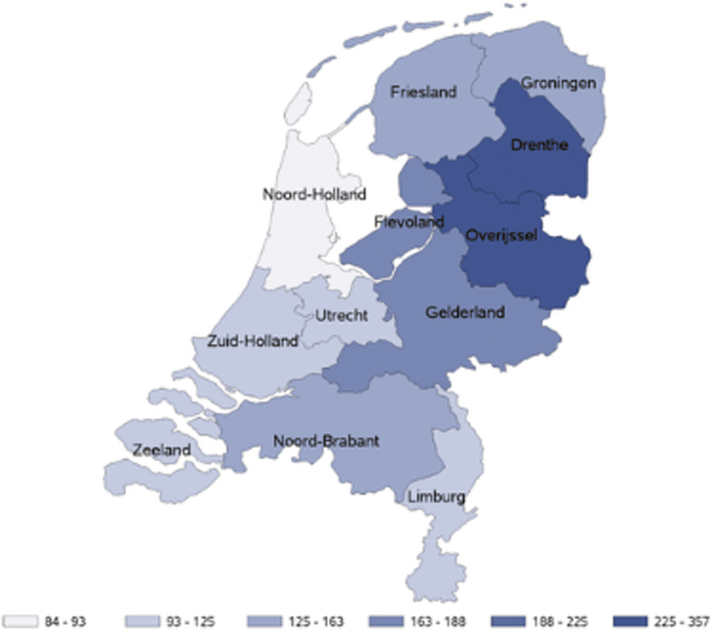
Incidence rates of GP-recorded LB in the Netherlands per 100,000 person-years presented per region over the entire study period.

**FIG. 3.**
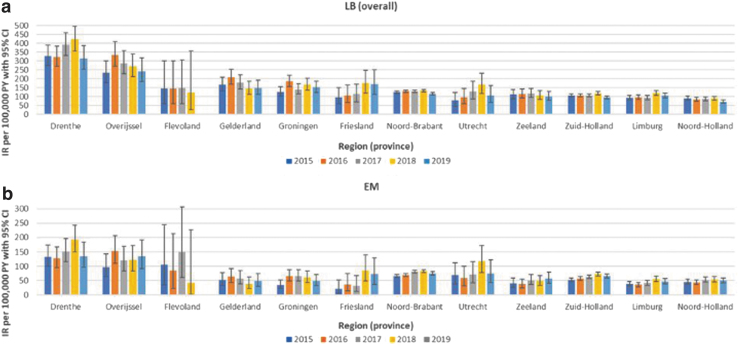
Incidence rates of GP-recorded LB and EM recorded in the Netherlands, stratified for region (province) and year: **(a)** LB overall; **(b)** EM. Note: for Flevoland 2019, IRs are not presented due to low numbers.

Incidence of LB over the entire study period was highest in the provinces of Drenthe and Overijssel: 357 (95% CI 330–386) and 276 (95% CI 248–306) per 100,000 person-years, respectively (Fig. 2); EM IRs over the entire study period were also highest in Drenthe (148 [95% CI 131–166]) and Overijssel (126 [95% CI 107–146]; data not shown). Annual IRs were consistent with these observations (Fig. 3).

**FIG. 4.**
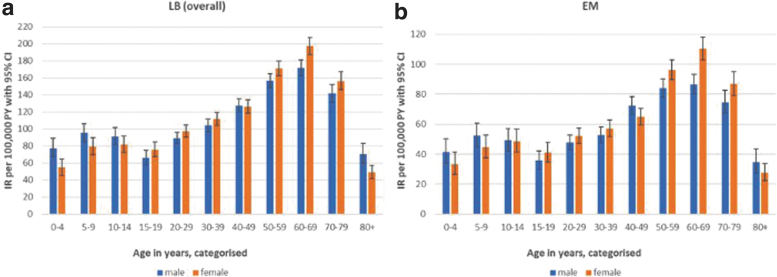
Incidence rates of GP-recorded LB and EM in the Netherlands, stratified for age and sex: **(a)** LB overall; **(b)** EM over the entire study period.

When stratified by age and sex (Fig. 4), LB incidence was slightly higher among individuals aged 5–9 and 10‒14 years, followed by a marginal drop among those aged 15‒19 years in both men and women. Incidence of LB then increased with age, with the highest IR per 100,000 person-years observed in the 60‒69 years age group (IR men: 172 [95% CI 163–182]; IR women: 197 [95% CI 187–207]), followed by a decline in older age groups. A similar pattern of IRs by age and sex was observed for EM and disseminated LB, although numbers were low for the latter group (data not shown).

Among immunocompromised subjects (*N* = 448,887; 16% of population at risk), the overall incidence of LB was 136 per 100,000 person-years (95% CI 131–141) and was significantly higher than among those who were not immunocompromised (IR: 118 [95% CI 116–120]). Additionally, subjects with a high SES had a significantly lower incidence of LB (IR: 95 [95% CI 92–98]) than subjects with a middle or low SES (IR medium: 132 [95% CI 128–136]; IR low: 135 [95% CI 131–139]). Similar differences were observed for the incidence of EM and disseminated LB stratified by immunocompromised status and SES (data not shown).

## Discussion

This large population-based cohort study provided updates regarding the annualized and group-specific IRs of LB events in the Netherlands among subjects registered with a GP. A consistent pattern of LB IRs was observed between 2015 and 2019 along with a bimodal age distribution characterized by IR peaks at 5‒14 and 60‒69 years of age in men and women. The highest IR was observed in 2018, which may be explained in part by the warm spring climate that year (Nature Today 2019), since warmer temperatures are projected to increase the range of suitable tick habitat (Beard et al. 2016).

The observed annualized and age-specific incidence of LB was consistent with previous work from Hofhuis et al. (2015a, 2015b, 2016) and van den Wijngaard et al. (2019); however, these previous Dutch studies used data from brief postal questionnaires sent to GPs, which can be prone to recall bias, instead of electronic health care records as used in the current study. Additionally, the design of the current study enabled categorization of LB manifestations as EM and disseminated LB, including LNB, LA, and other manifestations based on manual review of free text recorded by GPs. Our reported percentage of 3% of disseminated LB was similar to findings from a previous Dutch study (Hofhuis et al. 2015).

Incidence of LB and EM was geographically heterogeneous. In accordance with two other Dutch studies (Hofhuis et al. 2015, 2016), the incidence of LB and EM was highest in the provinces of Drenthe and Overijssel, which corresponds with tick presence through environmental risk mapping in the eastern forested, more humid regions (Wielinga et al. 2006, Gassner et al. 2011, Hofhuis et al. 2013, 2016, Swart et al. 2014, Garcia-Marti et al. 2018). These provinces are also inhabited by mammalian species (*e.g.*, deer and rodents) that create optimal habitats for sustaining the tick life cycle (Kenniscentrum reeën 2003, Ostfeld et al. 2006).

The high occurrence of LB in immunocompromised subjects is consistent with findings from Maraspin et al. (1999) and provides important new insight regarding subjects who may be at higher risk of LB, which could potentially support future interventions, such as vaccination, in vulnerable groups. However, information used to define immunocompromised status (*e.g.*, immunosuppressive therapy prescribed by specialists) was very likely underreported in the GP records as these patients are mainly treated by specialists; this may in turn have resulted in misclassification of these subjects, leading to potential underestimation of the increase in LB IR among immunocompromised individuals.

Our observation of decreased LB risk among subjects with a high SES contrasts with results from an ecological study of laboratory-confirmed LB cases in the United Kingdom that identified a correlation between high SES and higher incidence of LB (Tulloch et al. 2019). The investigators of the U.K. study suggested that their results may reflect leisure activities, leisure time, or access and attitudes to the countryside among individuals with higher SES.

The discrepancy between findings from the current study and the U.K. study may reflect cultural differences across European countries regarding demographic groups and pursuit of outdoor activity; however, it may also be influenced by varying national strategies on tick bite awareness and recent campaigns in the Netherlands (Stigas 2021), which have been shown to be effective interventional tools for protective behavior against ticks and LB (Beaujean et al. 2016). For example, tick bite awareness may have been higher among individuals with higher SES. Another explanation may be that people with higher incomes tend to live more in urban areas in the Netherlands. Information regarding these factors was not available, precluding examination of this hypothesis. Similar explorations of LB epidemiology in other European countries would be beneficial to put the results of our study into greater context.

We were able to use predefined algorithms to estimate LB events according to different manifestations, such as LA and LNB (National Institute for Public Health and the Environment [RIVM] 2013). However, use of data from health care databases, especially when LB was not directly related to the cause of visit, is subject to other challenges. Two studies reported that GPs regularly requested diagnostic tests (*i.e.*, serology) in cases of low LB suspicion; this diagnostic behavior may enhance overtreatment of LB (Botman et al. 2018, Vreugdenhil et al. 2020) and thereby deviate from national guidelines (National Institute for Public Health and the Environment [RIVM] 2013). It is plausible that cases with persistent positive responses to *B. burgdorferi sensu* lato in their serology test may not be indicative of an active infection (Kalish et al. 2001).

As a consequence, an artificially higher number of events in the current study may have been classified as confirmed or probable LB. Additionally, as noted, GPs are the gatekeepers within the Dutch health care system. Subjects with initial symptoms visit a GP but may be referred to a specialist in cases of uncontrolled disease or doubts regarding diagnosis. Patients with LB that were diagnosed, laboratory tested (*e.g.*, analysis of cerebrospinal fluid for LNB), or had antibiotics prescribed by specialists were only identified in GP records when GPs recorded communications with specialists. This limited information from specialists created challenges in confirming diagnoses and differentiating EM or disseminated LB manifestations from other conditions.

Future studies should focus on including further diagnostic results from specialists, which was beyond the scope of this article. As the presented IRs also included suspected and probable diagnoses, rates should be interpreted as a “worst-case scenario” for LB-related events and used primarily for identification of endemic areas and vulnerable populations. For EM, this can be disregarded, as its clinical appearance is used for diagnosis in clinical practice without requiring laboratory confirmation. Sensitivity analyses in which suspected events were excluded from the estimates for disseminated LB, which had a substantial proportion of suspected events, did not alter observed patterns. Furthermore, some of our results should be interpreted with caution due to low numbers resulting in less precise estimates; examples include stratified data for disseminated LB or those in provinces with few inhabitants (*e.g.*, Flevoland).

In conclusion, our study represents an updated, focused population-based assessment of LB incidence in the Netherlands. We demonstrated a consistent pattern of annual incidence of LB events between 2015 and 2019. High incidence of LB was identified in subjects diagnosed in the provinces of Drenthe and Overijssel. This study provides new insights into vulnerable groups that may be at higher risk of LB, such as individuals with lower SES or those who are immunocompromised, which should be further examined in the future.

## Supplementary Material

Supplemental data

Supplemental data

Supplemental data

Supplemental data

Supplemental data

## Prior Presentation

These results were previously presented at the 38th International Conference on Pharmacoepidemiology and Therapeutic Risk Management (ICPE; Copenhagen, Denmark; August, 24–28, 2022) and can be accessed at https://www.eventscribe.net/2022/ICPE/fsPopup.asp?efp=RExFSExBREsxMjMyMg&PosterID=494332&rnd=7.337105E-02&mode=posterinfo

## Data Sharing Statement

Data are available upon reasonable request at the PHARMO Institute. Requests for sharing study data must be made on specific grounds, either (1) with the aim of corroborating the study results in the interest of public health or (2) in the context of an audit by a competent authority. Sufficient information needs to be provided to confirm that the request is made for one of the above-mentioned purposes, including a sound justification and, in case of a request with a view to corroborate study results, a protocol on the research for which the data will be used or a plan for quality control checks, as applicable.
