# Supplementary material for: Incidence of Lyme Borreliosis in the Dutch General Practice Population: A Large-Scale Population-Based Cohort Study Across the Netherlands Between 2015 and 2019
Source: Vector Borne Zoonotic Dis. 2023 Apr 12;23(4):230–6. doi: 10.1089/vbz.2022.0048 (PMC10122225; doi:10.1089/vbz.2022.0048)
Supplement: Supplemental data [file Supp_TableS1.docx]

# Supplementary Table 1: Case definitions for LB and its manifestations in the PHARMO GP Database

| **Level of confirmation** | **LB** | **EM** | **LNB** | **LA** | **Other manifestations** |
| --- | --- | --- | --- | --- | --- |
| Suspected cases | An ICPC code for LB (ICPC A78.05)  **OR**  Any LB manifestation (ie, LB, EM, LNB, LA, or other manifestation) registered as free text* in GP notes | EM diagnosis registered as free text* in GP notes (note: no specific ICPC code available for EM) | LNB diagnosis registered as free text* in GP notes (note: no specific ICPC code available for LNB) | LA diagnosis registered as free text* in GP notes (note: no specific ICPC code available for LA) | ACA, Borrelial lymphocytoma, LC, or multiple EM diagnosis registered as free text* in GP notes (note: no specific ICPC codes available for these manifestations) |
| Probable cases | Suspected case  **AND**  Any ordered laboratory test results for LB (**Supplementary Table 2**)  **OR**  Receiving LB appropriate antibiotics (doxycycline, amoxicillin, azithromycin, ceftriaxone, benzylpenicilline) within ± 14 days of index date, except if it concerns 1-day prophylactic treatment or the linked diagnosis is respiratory disease (ICPC code chapter R), otitis media (ICPC code H70-H74), or urinary tract infection (ICPC code U71). | Suspected case  **AND**  Any ordered laboratory test results for LB (**Supplementary Table 2**)  **OR**  Receiving EM appropriate antibiotics (doxycycline, amoxicillin, azitromycin) within ± 14 days of index date, except if it concerns 1-day prophylactic treatment or the linked diagnosis is respiratory disease (ICPC code chapter R), otitis media (ICPC code H70-H74), or urinary tract infection (ICPC code U71).  NOTE: EM usually does not require laboratory confirmation and is based on clinical appearance in clinical practice. | Suspected case  **AND**  Receiving LB or EM appropriate antibiotics (doxycycline, amoxicillin, azithromycin, ceftriaxone, benzylpenicilline) within ± 14 days of index date, except if it concerns 1-day prophylactic treatment or the linked diagnosis is respiratory disease (ICPC code chapter R), otitis media (ICPC code H70-H74), or urinary tract infection (ICPC code U71).  **OR**  A positive serology in CSF as recorded by a GP.  NOTE: Tests for antibodies against B. burgdorferi in the spinal cord and cerebrospinal fluid is often used to diagnose neuroborreliosis. These tests are performed by a neurologist and not by a GP. The GP Database only contains the results of these tests if recorded actively by the GP. | Suspected case  **AND**  Receiving LB or EM appropriate antibiotics (doxycycline, amoxicillin, azithromycin, ceftriaxone, benzylpenicilline) within ± 14 days of index date, except if it concerns 1-day prophylactic treatment or the linked diagnosis is respiratory disease (ICPC code chapter R), otitis media (ICPC code H70-H74), or urinary tract infection (ICPC code U71). | Suspected case  **AND**  Receiving LB appropriate antibiotics (doxycycline, amoxicillin, azithromycin, ceftriaxone, benzylpenicilline) within ± 14 days of index date, except if it concerns 1-day prophylactic treatment or the linked diagnosis is respiratory disease (ICPC code chapter R), otitis media (ICPC code H70-H74), or urinary tract infection (ICPC code U71). |
| Confirmed cases | Suspected case  **OR**  Probable case  **AND**  Laboratory confirmation: a positive culture or PCR for B. burgdorferi (**Supplementary Table 2**) | Suspected case  **OR**  Probable case  **AND**  Laboratory confirmation: a positive culture or PCR for B. burgdorferi (**Supplementary Table 2**)  NOTE: EM usually does not require laboratory confirmation. | Suspected case  **OR**  Probable case  **AND**  Laboratory confirmation: a positive serology in CSF as recorded by a GP.  NOTE: Tests for antibodies against B. burgdorferi in the spinal cord and cerebrospinal fluid is often used to diagnose neuroborreliosis. These tests are performed by a neurologist and not by a GP. The GP Database only contains the results of these tests if recorded actively by the GP. | Suspected case  **OR**  Probable case  **AND**  Laboratory confirmation: a positive culture or PCR for B. burgdorferi (**Supplementary Table 2**) | Suspected case  **OR**  Probable case  **AND**  Laboratory confirmation:  a positive culture or PCR for B. burgdorferi (**Supplementary Table 2**) |

*An intense manual search of the free text in the medical record was conducted. Patients with, for instance, mentioning of ‘no EM’, ‘due to eczema’, or ‘ringworm’ were not included. ACA, acrodermatitis chronica atrophicans; CSF, cerebrospinal fluid; EM, erythema migrans; GP, general practitioner; ICPC, International Classification of Primary Care; LA, Lyme arthritis; LB, Lyme borreliosis; LNB, Lyme neuroborreliosis; PCR, polymerase chain reaction.
