# Supplementary material for: Incidence of Lyme Borreliosis in the Dutch General Practice Population: A Large-Scale Population-Based Cohort Study Across the Netherlands Between 2015 and 2019
Source: Vector Borne Zoonotic Dis. 2023 Apr 12;23(4):230–6. doi: 10.1089/vbz.2022.0048 (PMC10122225; doi:10.1089/vbz.2022.0048)
Supplement: Supplemental data [file Supp_TableS2.docx]

# Supplementary Table 2: Laboratory tests related to LB recorded in the PHARMO GP Database

| **WCIA code*** | **Description (in Dutch)** |
| --- | --- |
| 125 | Borrelia Burgdorferi IgA |
| 126 | Borrelia B. confirmation test |
| 127 | Borrelia Burgdorferi IgG |
| 128 | Borrelia Burgdorferi antistoffen (Lyme) |
| 129 | Borrelia Burgdorferi IgM |
| 2167 | Borrelia Burgdorferi C6 proteïne |
| 2226 | Borrelia Burgdorf. IgG (Westernblot) |
| 2227 | Borrelia Burgdorf. IgM (Westernblot) |
| 2404 | Borrelia B. index per total as. IgG/IgM |
| 2889 | Borrelia Burgdorferi IgG liquor |
| 2890 | Borrelia Burgdorferi C6 prot.antist.liq |
| 2891 | Borrelia Burgdorferi IgM liquor |
| 3101 | Borrelia Burgdorferi C6 prot.antist.kwant |
| 3102 | Borrelia Burgdorferi C6 prot.as.(1e,gprd) |
| 3103 | Borrelia Burgdorferi as.IgM kwantitatief |
| 3104 | Borrelia Burgdorferi IgM (1e mnstr,gprd) |
| 3434 | Borrelia Burgdorferi IgM kwantitatief |

*According to <http://aut.nhg.org/labcodeviewer/>. GP, general practitioner; LB, Lyme borreliosis; WCIA, Werkgroep Coördinatie Informatie Automatisering. See **Supplementary Table 1** for case definitions and how included laboratory tests contribute to the level of confirmation.
