# Supplementary material for: Incidence of Lyme Borreliosis in the Dutch General Practice Population: A Large-Scale Population-Based Cohort Study Across the Netherlands Between 2015 and 2019
Source: Vector Borne Zoonotic Dis. 2023 Apr 12;23(4):230–6. doi: 10.1089/vbz.2022.0048 (PMC10122225; doi:10.1089/vbz.2022.0048)
Supplement: Supplemental data [file Supp_TableS3.docx]

# Supplementary Table 3: Codes and definitions used to identify immunocompromised status

| **Diagnosis** | **ICPC / free text** | **Lab result** | **ATC** |
| --- | --- | --- | --- |
| End-stage renal disease | U99.01 - Renal impairment / renal insufficiency  **OR**  Registered as free text: ‘renal failure’, ‘dialysis’, ‘nephrotic syndrome’, or ‘kidney disease’ | eGFR <15 ml/min/1.73m^2^ | - |
| Malignant neoplasm | A79 - Malignity with unknown primary localization  B74 - Malignant neoplasm blood other  D74 - Malignant neoplasm stomach  D75 - Malignant neoplasm colon/rectum  D76 - Malignant neoplasm pancreas  D77 - Malig. neoplasm digestive system other/NOS  L71 - Malignant neoplasm musculoskeletal  N74 - Malignant neoplasm nervous system  R84 - Malignant neoplasm bronchus/lung  R85 - Malignant neoplasm respiratory, other  S77 - Malignant neoplasm of skin  T71 - Malignant neoplasm thyroid  U75 - Malignant neoplasm of kidney  U76 - Malignant neoplasm of bladder  U77 - Malignant neoplasm urinary other  W72 - Malignant neoplasm relate to preg.  X75 - Malignant neoplasm cervix  X76 - Malignant neoplasm breast female  X77 - Malignant neoplasm genital other (f)  Y77 - Malignant neoplasm prostate  Y78 - Malign neoplasm male genital other  **OR**  Registered as free text in GP episode: ‘cancer’, ‘malignant neoplasm’, or ‘chemotherapy’. | - | L01 - Antineoplastic agents |
| History of bone marrow transplant | Registered as free text: ‘bone marrow transplant’, ‘stem cell transplant’, or ‘stem cell therapy’ | - | - |
| Spleen anomalies | B87 – Splenomegaly  **OR**  Registered as free text: ‘spleen anomaly’, ‘splenomegaly’, ‘hypersplenism’ or ‘hypoplenism’ | - | - |
| AIDS | B90 - HIV-infection/AIDS  **OR**  Registered as free text: ‘AIDS’, ‘HIV’, or ‘seropositive’ | Positive test result for HIV | J05AR - Antivirals for treatment of HIV infections, combinations |
| Rheumatoid disorders | L88 - Rheumatoid/seropositive arthritis  K71 - Rheumatic fever/heart disease  **OR**  Registered as free text: ‘rheuma’ | - | M01 - Antiinflammatory and antirheumatic products |
| Immunodeficiency syndrome | T99.01 – Immunodeficiency  **OR**  Registered as free text: ‘immunodeficiency’ or ‘immunocompromised’ |  | L04 - Immunosuppressants |

ATC, Anatomical Therapeutic Chemical; GP, general practitioner; ICPC, International Classification of Primary Care.
