# Supplementary material for: Incidence of Lyme Borreliosis in the Dutch General Practice Population: A Large-Scale Population-Based Cohort Study Across the Netherlands Between 2015 and 2019
Source: Vector Borne Zoonotic Dis. 2023 Apr 12;23(4):230–6. doi: 10.1089/vbz.2022.0048 (PMC10122225; doi:10.1089/vbz.2022.0048)
Supplement: Supplemental data [file Supp_FigS1.docx]

# Supplementary Figure 1: Level of GP-recorded confirmation


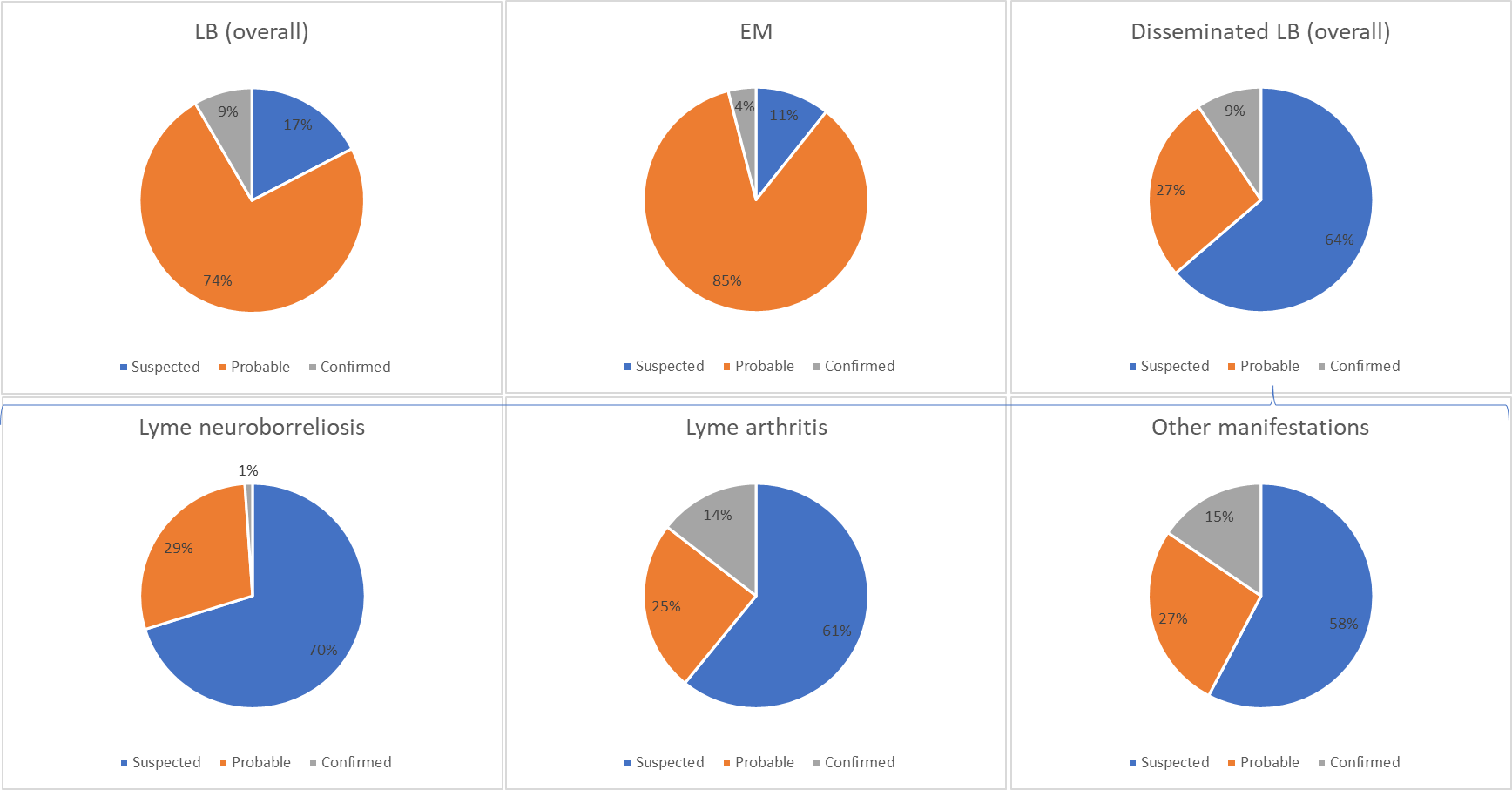


EM, erythema migrans; GP, general practitioner; LB, Lyme borreliosis.
