# Supplementary material for: Incidence of Lyme Borreliosis in the Dutch General Practice Population: A Large-Scale Population-Based Cohort Study Across the Netherlands Between 2015 and 2019
Source: Vector Borne Zoonotic Dis. 2023 Apr 12;23(4):230–6. doi: 10.1089/vbz.2022.0048 (PMC10122225; doi:10.1089/vbz.2022.0048)
Supplement: Supplemental data [file Supp_FigS2.docx]

# Supplementary Figure 2: Percentage of Lyme borreliosis events recorded by general practitioners in the Netherlands, by month (seasonal variability)
